# Comparison of GFR measurement with a two-blood sample technique using [^99m^Tc]Tc-DTPA vs. creatinine-based equations in potential kidney donors

**DOI:** 10.1590/2175-8239-JBN-2022-0105en

**Published:** 2022-11-28

**Authors:** José Pedro Carvalho, Andreia Marques, Fernando Abreu, Sophia Pintão

**Affiliations:** 1 Centro Hospitalar de Lisboa Ocidental, Hospital de Santa Cruz, Serviço de Medicina Nuclear, Carnaxide, Lisboa, Portugal.

**Keywords:** Glomerular Filtration Rate, Technetium Tc 99m Pentetate, Creatinine, Kidney Transplantation, Living Donor, Taxa de Filtração Glomerular, Pentetato de Tecnécio Tc 99m, Creatinina, Transplante de Rim, Doadores Vivos

## Abstract

**Introduction:**

Accurate determination of glomerular filtration rate (GFR) is crucial for selection of kidney donors. Nuclear medicine methods are considered accurate in measuring GFR but are not always easily available. The four-variable Modification of Diet in Renal Disease (MDRD4), Chronic Kidney Disease Epidemiology Collaboration (CKD-EPI), and Full Age Spectrum (FAS) formulas are common equations for estimating GFR and are recommended for initial assessment of kidney donors. The aim of this study was to evaluate the performance of these GFR estimation equations compared with technetium-99m diethylenetriaminepentaacetic acid ([^99m^Tc]Tc-DTPA) clearance.

**Methods:**

We compared GFR estimation by [^99m^Tc]Tc-DTPA clearance using a two-blood sample method with estimation by MDRD4, CKD-EPI, and FAS creatinine-based equations in a population of healthy potential kidney donors.

**Results:**

A total of 195 potential kidney donors (68.2% female; mean age 49 years, range 21–75 years) were included in this study. Mean [^99m^Tc]Tc-DTPA measured GFR (mGFR) was 101.5 ± 19.1 mL/min/1.73 m^2^. All three equations underestimated the GFR value measured by [^99m^Tc]Tc-DTPA (MDRD4: –11.5 ± 18.8 mL/min/1.73 m^2^; CKD-EPI: –5.0 ± 17.4 mL/min/1.73 m^2^; FAS: –8.3 ± 17.4 mL/min/1.73 m^2^). Accuracy within 30% and 10% of the measured GFR value was highest for CKD-EPI.

**Conclusion:**

The CKD-EPI equation showed better performance in estimating GFR in healthy potential kidney donors, proving to be a more accurate tool in the initial assessment of kidney donors. However, creatinine-based equations tended to underestimate kidney function. Therefore, GFR should be confirmed by another method in potential kidney donors.

## RESUMO

**Introdução:**

Determinar precisamente a taxa de filtração glomerular (TFG) é crucial para seleção de doadores de rim. Métodos de medicina nuclear são considerados precisos na medição da TFG, mas nem sempre estão facilmente disponíveis. As fórmulas *Modification of Diet in Renal Disease* de 4 variáveis (MDRD4), *Chronic Kidney Disease Epidemiology Collaboration* (CKD-EPI), e *Full Age Spectrum* (FAS) são equações comuns para estimar a TFG, sendo recomendadas para avaliação inicial dos doadores. Este estudo visou avaliar o desempenho destas equações de estimativa da TFG em comparação com o clearance do tecnécio-99m-ácido dietilenotriaminopentacético ([^99m^Tc]Tc-DTPA).

**Métodos:**

Comparamos a TFG por clearance de [^99m^Tc]Tc-DTPA usando um método com duas amostras de sangue com estimativa da TFG pelas equações MDRD4, CKD-EPI e FAS baseadas em creatinina em uma população de potenciais doadores saudáveis.

**Resultados:**

Incluiu-se 195 potenciais doadores de rim (68,2% mulheres; idade média de 49 anos, intervalo 21–75 anos). A TFG média medida por [^99m^Tc]Tc-DTPA foi 101,5 ± 19,1 mL/min/1,73m^2^. As três equações subestimaram o valor da TFG medida por [^99m^Tc]Tc-DTPA (MDRD4: –11,5 ± 18,8 mL/min/1,73 m^2^; CKD-EPI: –5,0 ± 17,4 mL/min/1,73 m^2^; FAS: –8,3 ± 17,4 mL/min/1,73 m^2^). A precisão dentro de 30% e 10% do valor da TFG medida foi maior para CKD-EPI.

**Conclusão:**

A equação CKD-EPI mostrou melhor desempenho na estimativa da TFG em potenciais doadores de rim saudáveis, revelando-se uma ferramenta mais precisa na avaliação inicial dos doadores. Entretanto, equações baseadas em creatinina tendem a subestimar a função renal. Portanto, a TFG deve ser confirmada por outro método em potenciais doadores.

## Introduction

Kidney transplantation is currently the preferred method for renal replacement therapy, as it is associated with improved quality of life and survival in end-stage renal disease (ESRD) patients, while being more cost-effective than dialysis in the long-term^1,2,3^. On the other hand, some studies suggest that there could be an increased long-term risk of ESRD in living kidney donors, especially in those with lower baseline glomerular filtration rate at donation, highlighting the need for careful assessment of kidney function in potential donors^4,5,6^.

Glomerular filtration rate (GFR) is considered the best index of overall kidney function^7^. The *Kidney Disease: Improving Global Outcomes* (KDIGO) guidelines suggest that candidates with a GFR of 90 mL/min/1.73 m^2^ or greater should be considered for donation, while individuals with GFR less than 60 mL/min/1.73 m^2^ should not be deemed suitable for donation. Regarding candidates with a GFR between these two values, eligibility should be based on an individualized approach incorporating demographic and health profile^8^.

Measurement of inulin clearance is the “gold standard” for the assessment of kidney function, but inulin’s availability and the costly, invasive and complex procedure limit its use in clinical practice^9^. Radioisotopic methods have shown to be reliable when compared to inulin clearance^10^. In our center, we use an *in vitro* technetium-99m diethylenetriaminepentaacetic acid ([^99m^Tc]Tc-DTPA) clearance rate quantification method to determine GFR, since most [^99m^Tc]Tc-DTPA elimination is by glomerular filtration, with no tubular secretion or reabsorption. However, nuclear medicine methods are only available at a limited number of institutions.

Estimation equations using endogenous filtration markers like serum creatinine have been used as an alternative for determining GFR in everyday practice and are recommended for initial assessment by the most recent KDIGO guidelines. The four-variable Modification of Diet in Renal Disease (MDRD4) formula is one of the most commonly used equations to estimate GFR. However, since it was developed using a population with impaired renal function, it tends to underestimate GFR in healthy individuals, which is an important limitation in potential kidney donors workup^11,12^. The Chronic Kidney Disease Epidemiology Collaboration (CKD-EPI) formula was developed using a population that included renal disease patients and healthy individuals, in order to provide a more accurate method in higher ranges of GFR^13^. Nevertheless, the use of these creatinine-based equations in individuals without renal function impairment is subject of debate. The Full Age Spectrum (FAS) equation was developed based on the concept of a population-normalized serum creatinine, with improved validity and continuity across the full age spectrum. It factors in correction for age and gender by including the mean or median serum creatinine value for age- and sex-specific healthy populations, derived from a healthy European population^14^.

However, some variables, such as muscle mass, diet, hepatic function and tubular secretion, may influence serum creatinine levels resulting in imprecision and inaccuracy^15^. This may lead to the rejection of suitable candidates with a falsely low estimated GFR, or even the acceptance of unsuitable candidates.

The aim of this study was to evaluate the performance of the most commonly used creatinine-based GFR estimation equations when compared with [^99m^Tc]Tc-DTPA clearance in healthy renal donors, in order to evaluate their validity in the assessment of living kidney donor candidates.

## Materials and Methods

In this retrospective study, 195 healthy potential kidney donors were evaluated at the Department of Nuclear Medicine of Centro Hospitalar de Lisboa Ocidental in Lisbon (Portugal) between January 2010 and March 2021. As part of the department’s pre-transplant assessment of potential living kidney donors, mGFR was determined using a renogram with [^99m^Tc]Tc-DTPA. GFR was estimated using three creatinine-based equations: MDRD4, CKD-EPI, and FAS.

### Measurement of Kidney Function

[^99m^Tc]Tc-DTPA clearance was measured using a two-blood sample protocol, based on the method described by Russel et al.^16^.

A regular dynamic renal study was performed with the administration of an intravenous bolus of 74–93 MBq (2–2.5 mCi) of [^99m^Tc]Tc-DTPA. Simultaneously with the preparation of the administered dose, a standard dose with the same activity (with a variation from the injected dose that did not exceed 5%) was also prepared. The standard dose was then submitted to a dilution process with distilled water, after which 1000 μL was pipetted into a micro tube and refrigerated for 24 hours.

Two blood samples were drawn from a contralateral vein after [^99m^Tc]Tc-DTPA administration. The timing of blood sampling was determined according to the GFR measured during the dynamic study by the Gates’ method^17^. If the GFR was ≥50 mL/min/1.73 m^2^, the samples were drawn at 1 and 3 hours after the radiopharmaceutical injection. If GFR was <50 mL/min/1.73 m^2^, the samples were drawn at 2 and 4 hours after. From each blood sample, plasma was separated by centrifugation and pipetted into a micro tube and refrigerated for 24 hours. For both blood samples, standard sample and background activity were measured in a well counter for 60 seconds, 24 hours after the radiopharmaceutical administration. The mGFR was then calculated using the formulas described by Russel et al.^16^.

### Kidney Function Estimations: Creatinine-Based Equations

Serum creatinine (sCr) was measured in our institution’s clinical laboratory using a Jaffé method traceable to isotope dilution mass spectrometry (IDMS). The sCr value closest to the renogram study was taken as reference. Patients without sCr results within 6 months of the renogram were excluded from this study.

Estimated GFR was calculated using the Modification of Diet in Renal Disease (MDRD4)^11^, the Chronic Kidney Disease Epidemiology Collaboration (CKD-EPI),^13^ and the Full Age Spectrum (FAS)^14^ equations.

### Statistical Analysis

Measurement data are presented as mean ± standard deviation (SD). All continuous variables had a normal distribution confirmed by the Kolmogorov-Smirnov test and the Shapiro-Wilk test. The association between eGFR and mGFR was assessed by correlation analysis using the Pearson coefficient of the logarithmic data. Performance results of eGFR equations are presented as bias, precision, and accuracy. Bias was defined as the difference between eGFR and mGFR. Precision was expressed as the root mean square error (RMSE). Accuracy was defined as the percentage of patients within 10% and 30% of mGFR. Paired t-tests and McNemar’s test were used to compare bias and accuracy, respectively. The Bland-Altman method was applied to evaluate the degree of agreement between eGFR and mGFR.

The statistical analysis was performed using the software SPSS version 20.0 (SPSS Inc., Chicago, IL, USA). Results were considered statistically significant when the *p* value <0.05.

## Results

A total of 195 potential kidney donors were included in this study. Characteristics of the studied population are shown in Table 1. Mean age was 49 years (full range 21–75 years), 133 individuals were female (68.2%) and 177 were Caucasian (90.8%). Mean serum creatinine value was 0.80 ± 0.16 mg/dL (0.46–1.50 mg/dL).

**Table 1.** Characteristics of study population (n = 195)

Age (years), mean (range)	49 (21–75)
Female, n (%)	133 (68.2)
Caucasian, n (%)	177 (90.8)
BMI (kg/m^2^), mean (range)	26.36 (18.62–39.61)
Serum creatinine (mg/dL), mean ± SD (range)	0.80 ± 0.16 (0.46–1.50)
mGFR (mL/min/1.73 m^2^), mean ± SD (range)	101.5 ± 19.1 (58–144)
eGFR MDRD4 (mL/min/1.73 m^2^), mean ± SD (range)	90.0 ± 17.9 (49–142)
eGFR CKD-EPI (mL/min/1.73 m^2^), mean ± SD (range)	96.5 ± 16.3 (52–136)
eGFR FAS (mL/min/1.73 m^2^), mean ± SD (range)	93.2 ± 18.6 (51–142)

The mean measured GFR using [^99m^Tc]Tc-DTPA (mGFR) was 101.5 ± 19.1 mL/min/1.73 m^2^. The mean estimated GFR (eGFR) using the MDRD4, CKD-EPI, and FAS equations were 90 ± 17.9 mL/min/1.73 m^2^, 96.5 ± 16.3 mL/min/1.73 m^2^, and 93.2 ± 18.6 mL/min/1.73 m^2^, respectively.

**Figure 1.**
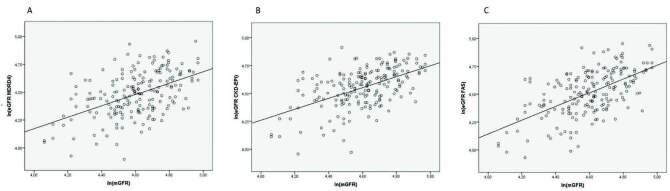
Scatter plot and linear regression between mGFR and the MDRD4 (A), CKD-EPI (B), and FAS (C) equations. (A) b = 0.73, r = 0.482, p < 0.001. (B) b = 0.84, r = 0.532, p < 0.001. (C) b = 0.80, r = 0.584, p < 0.001.

There was a significant correlation between each equation and mGFR (Figure 1). The FAS formula showed a slightly stronger positive linear correlation (r = 0.584, p < 0.001) than CKD-EPI (r = 0.532, p < 0.001) and MDRD4 (r = 0.482, p < 0.001).

Table 2 provides results for bias, precision, and accuracy of the eGFR equations. Overall, the CKD-EPI creatinine-based formula showed less bias and slightly better precision than both the MDRD4 and FAS equations. Additionally, accuracy within 30% and 10% of the mGFR were highest for CKD-EPI, followed by the FAS equation and MDRD4.

**Table 2.** Performance of the creatinine-based equations compared with mgfr by [^99m^Tc]tc-dtpa. rmse: root mean square error

Method	Mean difference from mGFR (95% CI)	RMSE (95% CI)	Accuracy (%) within	
			10% (95% CI)	30% (95% CI)
eGFR_MDRD4_	–11.5 (–14.1, –8.8)*	22.0 (20.0–24.4)	31.3 (24.9, 38.3)**	84.6 (78.8, 89.4)**
eGFR_CKD-EPI_	–5.0 (–7.5, –2.5)*	18.1 (16.4–20.1)	42.1 (35.0, 49.3)**	92.3 (87.6, 95.6)**
eGFR_FAS_	–8.3 (–10.8, –5.8)*	19.3 (17.5–21.4)	37.4 (30.6, 44.6)**	90.8 (85.8, 94.4)**

* p < 0.001, ** p < 0.05

**Figure 2.**
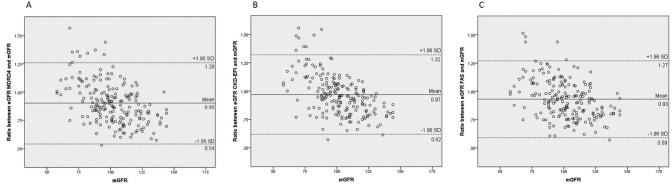
Bland-Altman plots of the MDRD4 (A), CKD-EPI (B), and FAS (C) equations. The solid line represents the mean difference between eGFR and mGFR, and the dashed lines represent the upper and lower limits of agreement with 95% confidence intervals.

Bland-Altman plots comparing mGFR with each equation are shown in Figure 2. In our study, we observed an increase in variability of the differences between each method and mGFR as the magnitude of the measurement increased. Thus, the ratio between methods was plotted against the reference method. All the equations underestimated the mGFR, with the CKD-EPI formula showing a closer relationship with the reference method.

## Discussion

An accurate assessment of kidney function in donor candidates is critical, for determining the function of not only the future graft, but also of the donor’s remaining kidney. In this study we investigated the performance of creatinine-based equations for the estimation of GFR in a population of potential kidney donors.

We found that all three creatinine-based equations tended to underestimate GFR when compared with the *in vitro* GFR measurement. This discrepancy may be a result of GFR-unrelated factors influencing serum creatinine concentration, such as body composition and diet.

Of all the eGFR equations, the CKD-EPI formula showed better performance. It showed less bias, slightly better precision, and was more accurate than the MDRD4 and FAS equations. These findings are consistent with previous reports^18,19,20,21,22^. On the other hand, the performance of the MDRD4 formula was subpar compared with the other estimation equations. Consequently, the MDRD4 equation is not recommended for estimating GFR in a presumably healthy population as is the case of potential kidney donors. The CKD-EPI creatinine-based formula, despite not being optimal, seems to be a more accurate estimating method.

These results highlight the need for a careful interpretation of GFR results obtained by these estimating equations in the assessment of healthy potential kidney donors. The significant underestimation of GFR values may lead to exclusion of candidates based on an incorrect estimation of kidney function. Therefore, we believe that the use of measuring methods for determining GFR is of particular importance in this context, especially when the estimated GFR falls under the 90 mL/min/1.73 m^2^. This is also in line with some of the current guidelines. The KDIGO guidelines suggest that the GFR should be confirmed by a measured GFR method, either using an exogenous filtration marker (such as [^99m^Tc]Tc-DTPA), measured creatinine clearance (mCrCl) or that GFR should be estimated by combining serum creatinine and cystatin C (eGFR_cr-cys_). Moreover, in patients with known asymmetry of kidney size or parenchymal, vascular, or urological abnormalities, GFR should be assessed by a radionuclide method in order to measure the contribution of each kidney to the global kidney function^8^. On the other hand, the European Renal Best Practice Guideline group only recommends the direct measurement of GFR in uncertain cases^23^.

Based on these recommendations, our institution’s transplantation protocol contemplates an initial assessment by a serum creatinine-based equation, which is posteriorly confirmed by measuring the clearance of [^99m^Tc]Tc-DTPA. Other confirmatory methods, such as mCrCl or eGFR_cr-cys_, may be used in centers without nuclear medicine methods available, although their accuracy compared to radioisotopic methods in this population should be evaluated in future studies, as well as their utility in the initial assessment of potential donors.

The main limitation of the current study was the small sample size analyzed, making it difficult to extrapolate the results to the broad spectrum of potential donors’ population. Therefore, further studies may be necessary. Additionally, another limitation lies in the fact that serum creatinine was not determined from a blood sample drawn on the day the renogram was performed. However, given that the population of our study was presumably healthy, we considered that there wouldn´t be a significant difference in the serum creatinine value in a span of 6 months.

In conclusion, the measurement of clearance of an exogenous substance remains the most reliable method to determine kidney function in healthy individuals, such as potential kidney donors. Our findings support the use of the CKD-EPI creatinine-based formula to estimate GFR for initial assessment, as it provides the most reliable results among the studied equations. However, as creatinine-based estimation equations tend to underestimate renal function, the findings should be interpreted with caution. If possible, the estimated GFR should be confirmed by a measuring method, especially in uncertain cases, so that potential kidney donors are not incorrectly excluded.
